# Protein-C deficiency presenting as pulmonary embolism and myocardial infarction in the same patient

**DOI:** 10.1186/1477-9560-11-19

**Published:** 2013-10-01

**Authors:** Syed Maqbool, Vishal Rastogi, Ashok Seth, Satbir Singh, Vijay Kumar, Arif Mustaqueem

**Affiliations:** 1 Invasive Cardiology, Fortis Escorts Heart Institute, New Delhi 110025, India; 2 Cardiovascular Sciences, Fortis Escorts Heart Institute, New Delhi 110025, India; 3 Fortis Escorts Heart Institute, New Delhi 110025, India; 4 Noninvasive Cardiology, Fortis Escorts Heart Institute, New Delhi 110025, India

## Abstract

Plasma protein-C exerts anticoagulatory effects by inactivating factors V and VIII. Hereditary protein C deficiency is transmitted as an autosomal dominant disorder. Homozygous individuals usually develop purpura fulminans as newborns; heterozygous protein C-deficient individuals are at increased risk for venous thrombosis and pulmonary embolism. However, arterial thrombosis occurring as a result of congenital protein-C deficiency is still controversial. We describe a young patient with heterozygous protein-C deficiency who experienced both pulmonary embolism as well as myocardial infarction due to thrombotic occlusion without underlying major risk factors. Acute myocardial infarction in young without underlying major risk factors may be evaluated for protein c deficiency.

## Introduction

Protein C is a 62-kD, vitamin K-dependent glycoprotein synthesized in the liver. It circulates in the blood as an inactive zymogen at a concentration of 4 μg/ml. Its activation into the serine-protease enzyme activated protein C (aPC), is catalyzed by thrombin when it is bound to the endothelial glycoprotein thrombomodulin [1,2]. aPC exerts its anticoagulant activity primarily through inactivation of coagulation factors Va and VIIIa, which are required for factor X activation and thrombin generation. The catalytic activity of aPC is greatly enhanced by the vitamin K-dependent cofactor protein S [3]. A deficiency of protein C disturbs the delicate balance between procoagulant and anticoagulant proteins and engenders a prothrombotic state. Cardinal manifestation of protein-C deficiency is venous thromboembolism. However, there are several case reports of arterial stroke [4,5] and myocardial infarction [6-11] occurring in young adults with congenital protein-C deficiency; but the results of larger studies are conflicting [12-16] and the existence of an association between protein-C deficiency and arterial thrombosis remains controversial. We present a case who had pulmonary thromboembolism 3-years back and now presented with acute myocardial infarction without any underlying major risk factors for coronary artery disease.

## Case presentation

**Figure 1:**
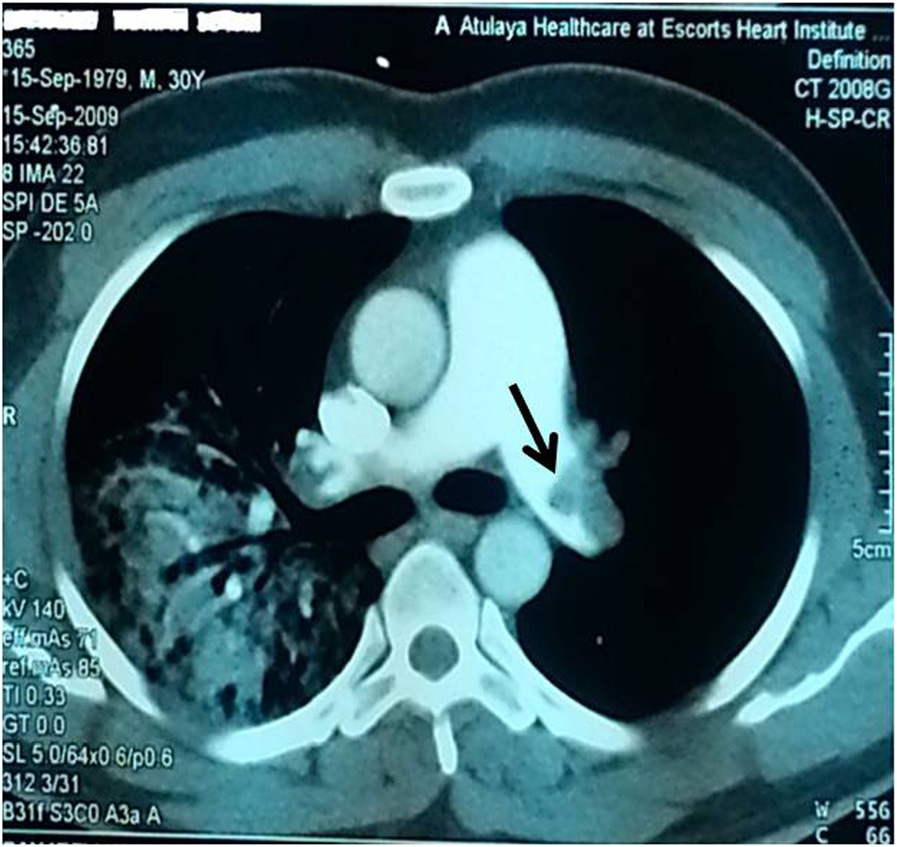
CT angio showing thrombus in the left pulmonary artery (black arrow) and lung infarct on the right side (white arrow).

**Figure 2:**
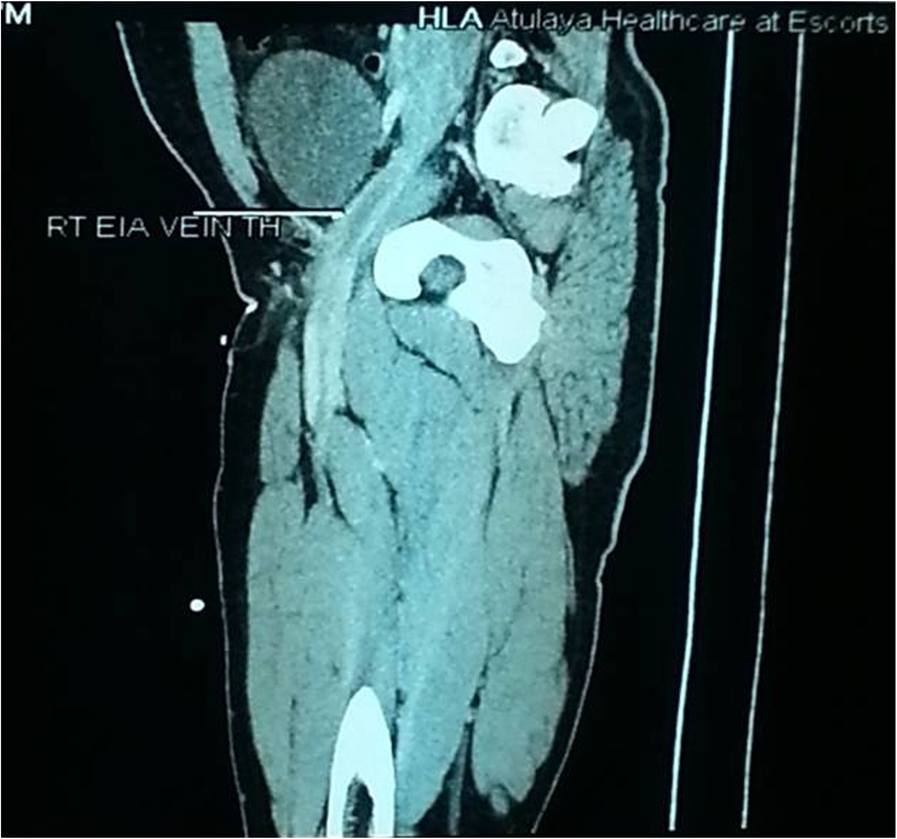
Thrombus in the right external iliac vein (long arrow).

**Figure 3:**
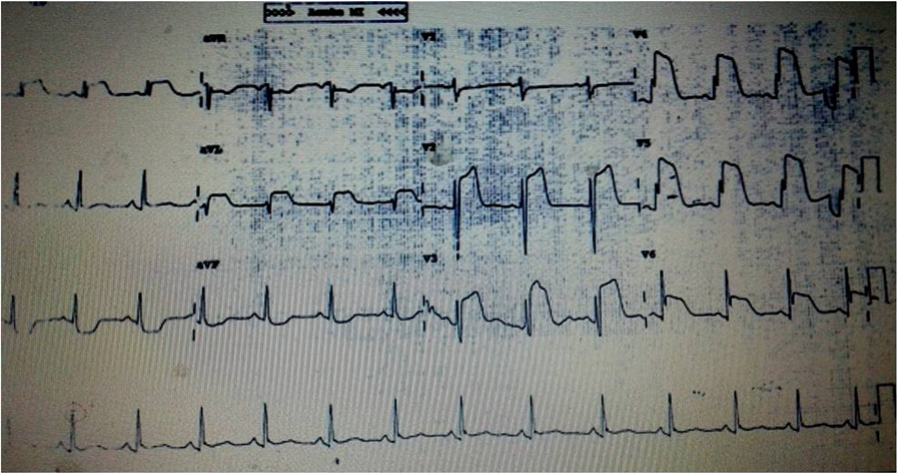
Electrocardiogram showing Extensive anterior wall myocardial infarction.

**Figure 4:**
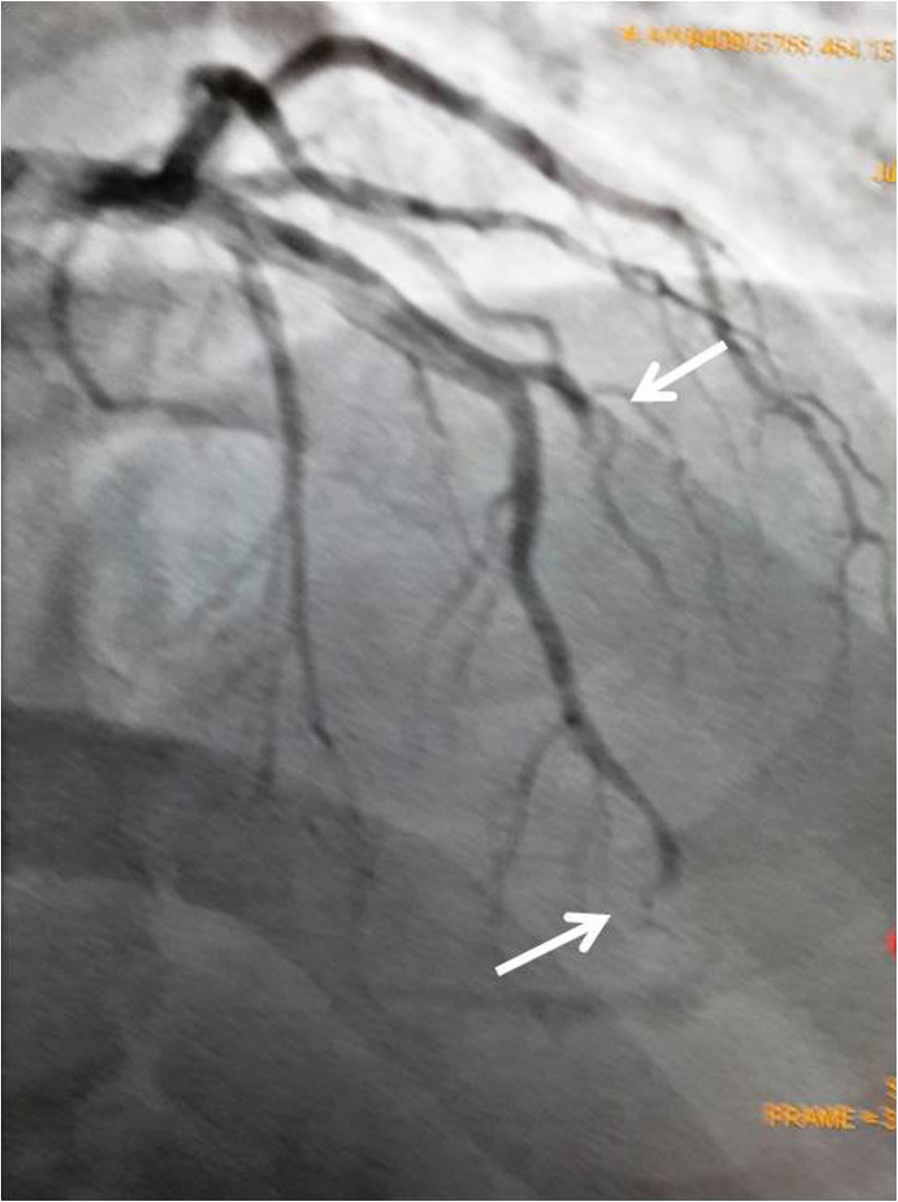
RAO cranial view of angiogram showing distal LAD and proximal D2 cut off (white arrows).

**Figure 5:**
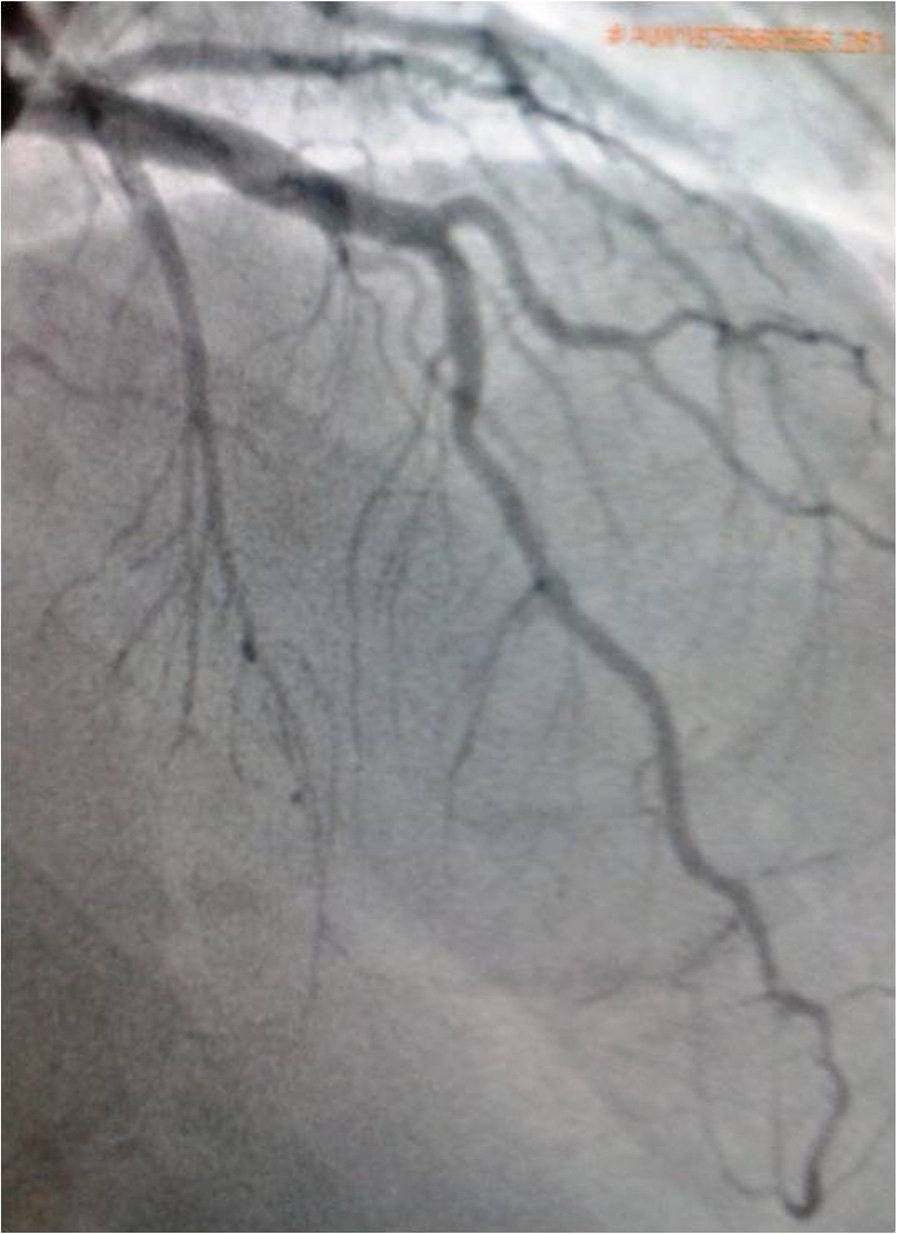
RAO cranial view post thrombosuction showing fully patent LAD and D2 without any underlying atherosclerosis.

**Figure 6:**
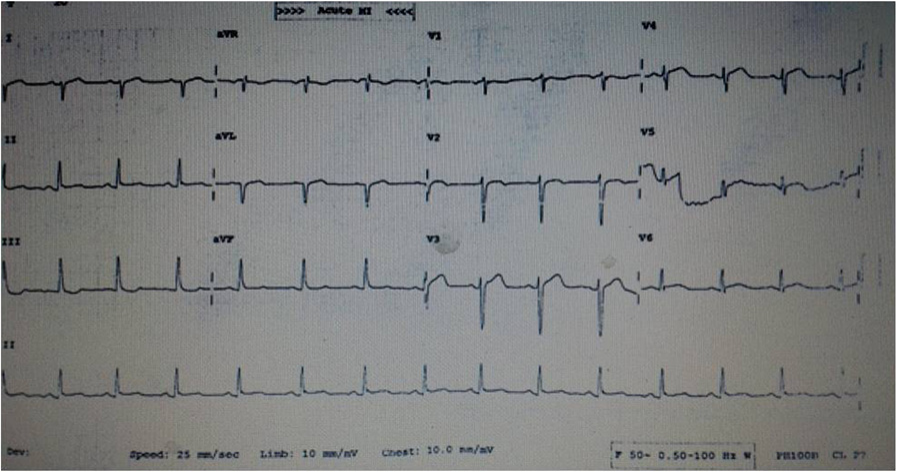
Electrocardiogram after thrombosuction.

Our patient is a 37-years old male who had presented in 2009 with acute severe dyspnea and retrosternal chest pain; his Electrocardiogram showed sinus tachycardia with s1 q3 t3 pattern; Echo showed mildly dilated right ventricle; D-dimer was >15micrograms/ml; CT angiography showed acute pulmonary thromboembolism (Figure 1) and right lower limb external iliac, common femoral and popliteal vein deep venous thrombosis (Figure 2). He was managed with heparin and discharged on oral anticoagulation. Though, erratic with his anticoagulation, he nevertheless was doing well till morning of 26^th^ Jan 2013 when he developed severe retrosternal chest pain radiating to both arms. He was brought to hospital in an hour and his Electrocardiogram showed extensive anterior wall myocardial infarction (Figure 3). He was directly taken to the lab where the angiogram showed distal left anterior descending and proximal diagonal-2 cut off with thrombus (Figure 4). Thrombosuction was done and TIMI- 3 flow was restored (Figure 5). The underlying arteries were clean with no evidence of atherosclerosis. His electrocardiogram post thrombosuction reverted almost to normal (Figure 6). He had no major risk factors for coronary artery disease (Table 1). Patient was restarted on anticogulation and sent home on fourth day. His investigations showed normal blood counts, chemistry, thyroid and lipid profile. His coagulation profile showed normal results for homocysteine, antithrombin, protein S,prothrombin gene mutation, factor V Leiden and antiphospholipid antibody syndrome (Table 2). His protein-C activity was low 42%. Transthoracic Echocardiogram was normal and PFO was ruled out by Transesophageal Echocardiogram.

**Table 1:** CAD risk factors

Blood sugar	
Fasting	86
PP	110
HbA1c	5.1%
LDL	96 mg/dl
TG	110 mg/dl
BMI	20
B.P.	120/80 mmHg
Non-smoker	

**Table 2:** Coagulation profile

**Test**	**Patient value**	**Reference range**
**Protein-C activity, plasma**	42%	67–195%
**Protein-S activity, plasma**	99%	77–143%
**Antithrombin activity, plasma**	94%	70–122%
**Homocystiene, serum/plasma**	11.25 micromoles	5.46 -16.20 μmoles
**Factor V leiden mutation**	Not Detected	
**Antiphospholipid antibodies**		
*Lupus Anticoagulant*		
DRVVT	36 sec	32.8–48.9 sec
DRVVT T: C Ratio	0.89	0.82–1.22
*Anticardiolipin Antibody*		
*(Aeskulia-Cardiolipin-GM) Elisa*		
Anticardiolipin (IgG)	1.14 GPL	POSITIVE >15 .0 GPL
Anti Beta 2 Glycoprotein 1 (IgG)	1.10 GPL	POSITIVE >15.0 GPL

## Discussion

Protein-C deficiency by plasma level alone is found in 1 in 200 to 1 in 500 persons in the general population [17,18]. However, many affected individuals remain asymptomatic throughout life. The cardinal clinical manifestation of heterozygous protein C deficiency is venous thromboembolism [19,20]. There are several case reports of arterial thrombosis including stroke, myocardial infarction and peripheral arterial thrombosis. However, the results of larger studies have reported against protein c deficiency causing arterial thrombosis. Among the so far reported 23-cases of myocardial infarction due to protein c deficiency only six had angiography done, 2 underwent coronary intervention, one received a stent and in other the obstruction could not be relieved. Rest of the patients were thrombolysed but coronaries remained totally occluded in 3 and partially in one [6]. In our patient just thrombosuction cleaned up the coronaries and post thrombosuction even mild plaquing was not seen anywhere in the coronary tree. Our patient had absolutely no major risk factors for coronary artery disease. Patent foramen ovale was ruled out by contrast TEE. These findings suggest that protein c deficiency may cause thrombus formation in the arterial system without any underlying atherosclerosis and point to the fact that young patients with acute myocardial infarction without underlying major risk factors for coronary artery diseasers may be evaluated for protein c deficiency. Protein-C deficiency can occur during acute thrombosis or when the patient is on anticoagulation and the only way to confirm protein-C deficiency in such cases is to check the family members or to stop anticoagulation for two weeks and to wait until acute stage is over. Our patient had recurrent thrombotic episodes so we preferred to check the family members rather than stopping the anticoagulation. His father showed a very low level of 26% and mother had a level of 46% of protein-C activity, but there was no family history of thrombosis. It is known that all patients with protein-C and/or S deficiency donot experience thrombotic events and that 0.3% of blood donors have low protein-C levels without any overt thromboembolic episodes [21].

## Conclusion

Protein-C deficiency can cause myocardial infarction in young adults with normal coronaries and without any underlying major risk factors for coronary artery disease. The message is that young patients with acute myocardial infarction with no major risk factors may be evaluated for protein c deficiency; however, it needs larger studies to be established.

## Informed consent

“Wriiten informed consent was obtained from the patient for the publication of this report and any accompanying images”.

## Competing interests

The authors declare that they have no competing interests.

## Authors’ contributions

SM conception data accumulation. AS & VR analysis. SS & AM echocardiography. VK editing. All authors have read and approved the final version of the manuscript.
